# Supplementary material for: Implementation of EHMRG Risk Model in an Italian Population of Elderly Patients with Acute Heart Failure
Source: J Clin Med. 2022 May 25;11(11):2982. doi: 10.3390/jcm11112982 (PMC9181787; doi:10.3390/jcm11112982)
Supplement: Supplementary file 1 [file jcm-11-02982-s001.zip › jcm-1733105-supplementary.pdf]

Implementation of EHMRG Risk Model in an Italian Population of Elderly Patients with Acute Heart Failure

Supplementary Materials

Table S1. Differences between surviving and non-surviving patients.

**Clinical Variables**	**Surviving** (*n* = 394)	**Non-Surviving** (*n* = 45)	**p**
Age, years, (±SD)	84.3 (±7.65)	87.1 (± 8.11)	0.024
Males (*n*, %)	161 (36.7%)	19 (4.3%)	0.861
NYHA class, [IQR]	2 [1]	3 [1]	0.0001
Length of hospitalization, days, [IQR]	11.2 (±6.92)	9.51 (±7.85)	0.121
BNP on admission, pg/ml, [IQR]	600.5 [805]	560.5 [846]	0.001
SBP, mmHg, (±SD)	127.5 (±28.1)	128.0 (±28.2)	0.001
HR, bpm, (±SD)	89.4 (±24.6)	90,4 (±23.9)	0.104
SpO2, %, (±SD)	91.8 (±7.3)	92,0 (±7.07)	0.135
Creatinine, mg/dl, (±SD)	1.6 (±1.0)	1.45 (±0.99)	0.006
Potassium, mmol/l, (±SD)	4.00 (±0.69)	4.04 (±0.65)	0.098
Out of range Potassium, (*n*, %)	180 (41.1%)	74 (53.6%)	0.016
Troponin, ng/ml, [IQR]	0.05 [0.10]	0.05 [0.11]	0.172
Increased Troponin, (*n*, %)	204 (46.5%)	63 (45.7%)	0.0001
ED arrival by ambulance, (*n*, %)	284 (64.7%)	83 (60.1%)	0.023
Active cancer, (*n*, %)	79 (18.0%)	1 (0.2%)	0.003
Metolazone use, (*n*, %)	9 (2.1%)	3 (0.72%)	0.088
EHMRG, [IQR]	69 [98.4]	60.8 [99,3]	0.0001
EHMRG Class, [IQR]	5 [2]	5 [3]	0.001

Table S2. Cox regression analysis.

Panel A: Whole sample (n = 439)					
**Variable**	**B**	**HR**	**95%CI**		**p**
			**Lower**	**Upper**	
EHMRG Category	1.046	2.85	1.64	4.98	0.0001
NYHA Class	1.044	2.84	1.78	4.54	0.0001
BNP at the Admission	0.000	1.00	1.00	1.00	0.297
Sex	-0.121	0.89	0.43	1.81	0.740
Panel B: Seven days (n=138)					
**Variable**	**B**	**HR**	**95%CI**		**P**
			**Lower**	**Upper**	
EHMRG Category	0.977	2.66	1.36	4.97	0.004
NYHA Class	0.928	2.53	1.13	5.64	0.023
BNP at the Admission	0.000	1.00	1.00	1.00	0.156
Sex	-0.626	0.53	0.18	1.56	0.252

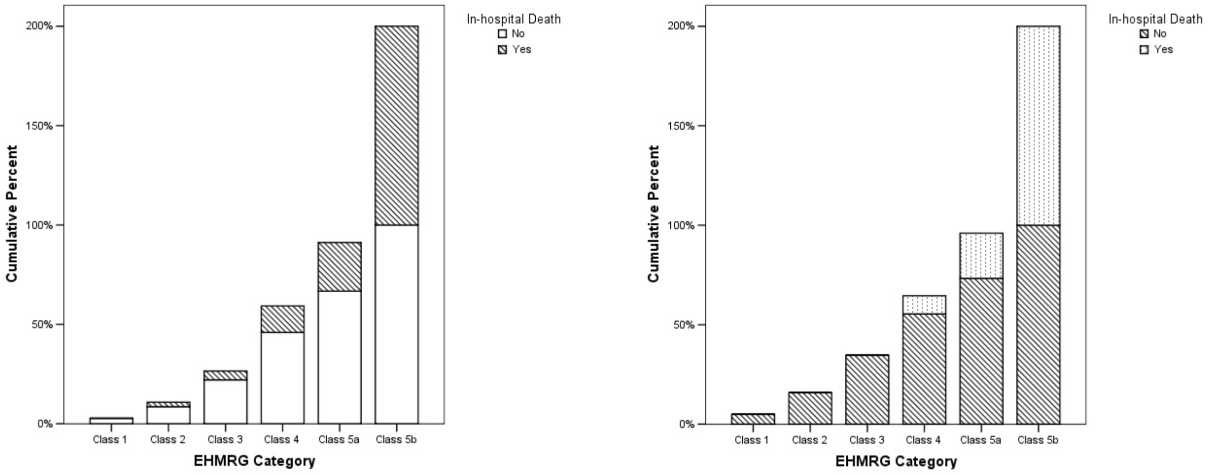

Panel A. Whole sample (n=439) Panel B. 7-days (n=138)

Figure S1. Cumulative percentage of in-hospital deaths according to EHMRG ($p < 0.0001$).

Filename: jcm-1733105-supplementary.docx
Directory: E:\5.25\sensors-1733280
Template: C:\Users\MDPI\AppData\Roaming\Microsoft\Templates\Normal.dotm
Title:
Subject:
Author: Lorenzo Falsetti
Keywords:
Comments:
Creation Date: 3/15/2022 7:20:00 PM
Change Number: 6
Last Saved On: 5/25/2022 3:01:00 PM
Last Saved By: MDPI-52
Total Editing Time: 16 Minutes
Last Printed On: 5/25/2022 6:32:00 PM
As of Last Complete Printing
Number of Pages: 2
Number of Words: 313 (approx.)
Number of Characters: 1,549 (approx.)
